# The Institutional Library

**Published:** 1915-01-09

**Authors:** 


### THE INSTITUTIONAL LIBRARY

### Quain Up to Date.

Quain's Elements of Anatomy. Eleventh Edition. Volume II., Part II.: Splanchnology. By J. Symington, M.D., F.R.S. With 349 illustrations. (London: Longmans, Green and Co. 1914. Price 10s. 6d.)

It used to be a common notion among medical students that anatomy "did not change much," and that the books of the preceding generation would serve excellently, and that good cash could be saved by the purchase of second-hand text-books. With physiology books this was too dangerous a game: new "explanations" sprouted every year. Nowadays it seems that anatomy is also undergoing continual metamorphosis. Changes in nomenclature are not the only signs of progress; considerable "structural alterations" have been called for as the result of research. Of course "Quain's Anatomy" has sacrificed not one iota of its old-established claim to precedence over other anatomical text-books. In the old days it was only the very superior "higher diploma" lads who read "Quain," perhaps now even the "Conjoint boys" dip into it, but the "Fellowship men" still "swear" by it. The present volume is the second part of volume ii. in the eleventh edition. It deals with the topographical anatomy and morphology of all the viscera. Professor Johnson Symington, of the Queen's University, Belfast, and one of the three distinguished editors of the complete work, is responsible for this volume. Needless to say, the traditions of "Quain" for thoroughness and detail are carried on. A good deal of re-writing has been required, and new figures are fairly common. A long new section, describing the peritoneum, has been written by Mr. P. T. Crymble; this is illustrated by a large number of very fine diagrams, and forms a classical monograph on this complicated membrane. In the section on the delimitation of the abdomen the well-known charts of Dr. Christopher Addison are included.

### Bacteriology for Nurses.

Elementary Bacteriology for Nurses. By G. Norman Meachen, M.D. Lond., M.R.C.P. Illustrated. (London: The Scientific Press, Ltd. Price 2s.)

There can no longer be any hesitation in describing bacteriology as holding a position among the medical details of which nurses are expected to take a certain cognisance. Nurses are taught nowadays many things which are much more specialised than the elements of bacteriology, and which are less often required to be brought into practical application than a knowledge of the ways and workings of "germs." In the matter of text-books on the subject of bacteriology suitable for the use of nurses Dr. Meachen is not the first in the field, but we can truthfully say that in preparing this subject for the digestion of nurses he has shown himself competent in the selection and arrangement of those portions which will be of the most service to them and to the medical men under whom they are instructed. Readers of the *Nursing Mirror* will recall the interest with which they read Dr. Meachen's lectures as they appeared serially in that journal, and it is in deference to requests for a more convenient and more permanent record of his lectures that this text-book has been published. In the course of eighteen lectures the author covers the field of elementary bacteriology very suitably, and incidentally touches on many little side issues which serve to add greatly to the interest and pleasure with which the book will be read. The list of authorities quoted is scarcely needed in a book of this class, but the full index will certainly be appreciated.

### The General Practitioner and Syphilis.

Genito-Urinary Diseases and Syphilis. By E. G. Ballenger, M.D., assisted by Omar F. Elder, M.D. Second Edition, Revised. 109 illustrations. (London: Butterworth and Co. 1914. Price 16s. net.)

The second edition of this American text-book of venereal and genito-urinary diseases has been revised so thoroughly that it may claim to be considered up to date. As a practical working manual it has good points that will commend it to the general practitioner, while the senior student will not find it difficult to gain a useful knowledge of these diseases from its pages; in fact, a series of interpolated summaries is provided chiefly for his benefit. The authors have used a direct and incisive style that has enabled them to cover the wide field indicated by the title within the limits of a not too bulky volume. Two special chapters explanatory of the Wassermann reaction have been contributed by Dr. J. E. Paulin, and in relation to gonorrhœa the complement fixation test is advocated as the most accurate method of detecting latent disease. Of the new matter mention may be made of the treatment of gonorrhœa in its early stage by sealing argyrol in the urethra by means of a collodion scab on the meatus; this is described with practical detail and a good case made out for it. As a test for gauging the functional capacity of the kidney prominence is given to the phenol-sulphone-phthalein test of Geraghty; the technique of this might well have been given in fuller detail, and the colorimeter itself should have been described. The etiology of hypertrophy of the prostate is discussed at some length, with the conclusion that this condition results from the long-continued irritation of a mild toxin produced by a growth of attenuated organisms. In the treatment of syphilis neosalvarsan claims as much attention as salvarsan, and the authors preface this chapter with a warning to medical men that the charges made should be for the cure rather than for each injection. Dr. McDonagh's work on the life history of the spirochæte is included in this section, as well as much other recent research. A few errors remain uncorrected, especially in the spelling of proper names, and one of the figures is upside down, while fig. 66 is surely not worth a whole page.

### The Training of a Sanitary Inspector.

Practical Tropical Sanitation: A Manual for Sanitary Inspectors. By W. Alex. Muirhead, Staff Sergeant R.A.M.C. Illustrated. (London: John Murray. 1914. Price 10s. 6d. net.)

It is not often that one has to deal with a book which so suitably fulfils the purpose for which it was written as this new manual on sanitation. The author, who is a staff sergeant in the Royal Army Medical Corps and an assistant instructor at the School of Army Sanitation at Aldershot, gained his practical experience in tropical sanitation in the West African command. He is obviously a man who, knowing his own job, is in no doubt as to what would-be sanitary inspectors ought to know. As a class teacher he should be excellent; as a writer of a practical manual he has achieved a creditable success. That there is room for such a book as this may be taken for granted; for one thing it brings together in a handy form those details of sanitation in the tropics which are only to be found scattered in various books mostly intended for medical officers and couched in technical language. This volume is especially compiled to meet the needs of candidates for tropical sanitary appointments, and includes all those matters germane to the training of a sanitary inspector. The text is concise and to the point, and although one comes across a few slightly ambiguous sentences, the matter is valuable enough to demand careful reading on the part of the student if he is to extract all the practical teaching that lies within. The assumption is made that the reader is without much special sanitary training, for it is seldom that experienced inspectors from the old country, on account of home ties, take up tropical appointments. Hence the book covers a wide field and elementary matters are not overlooked, but the author has kept constantly in view the fact that he is writing for men who are or will be working in tropical lands. To pick out some points for special mention, the author may be congratulated on his introductory chapters and on those dealing with disinfection and water supplies. Throughout the book the illustrations—both plates and diagrams —are of real assistance, and it is pleasing to note that where a diagram in itself explains all that is necessary, the author does not waste words in going over the points again in the text. The important subject of disinfectants is handled more clearly than in many more pretentious books, and the chapter on this branch of the subject includes most things, from chemicals to the Clayton apparatus and high-pressure steam machines; even the delusion of the antiseptic power of "antiseptic" soaps comes in for mention—a very wise and practical attention. The section on mosquitoes is exhaustive, and the accompanying numerous illustrations, including an ingenious diagram of the connection between man and the mosquito in the case of malaria, are clear and helpful. It only remains to note that the author has been well served by the publisher—the printing is excellent—but one may ask if top gilt edges are not unnecessary for a book of this description. Of misprints only one has been noticed—a figure on page 265 which should read 29.10.

### An Old Favourite on Nursing.

General Nursing. By Eva C. E. Lückes. New and Revised (Ninth) Edition. (London: Kegan Paul, Trench, Trübner and Co. 1914. Pp. 347. Price 5s. net.)

This ever-popular handbook has now been in existence for thirty years, testimony, if any were needed besides the fact that nine editions have appeared, to the soundness of its principles and the skill with which they have been adapted to changing times and ideas. Thirty years ago Listerian surgery had only just been universally accepted in London as the sure doctrine of surgical salvation; and most of the surgeons then on the in-patient staffs of the London hospitals neither understood it nor in their secret consciousness believed in it. In the present re-issue Miss Lückes has been assisted on certain special subjects by several gentlemen of the London Hospital officers. Thus Professor Leonard Hill and Dr. R. A. Rowlands have written a chapter on ventilation, and Professor Bulloch has brought up to date the sections dealing with fever nursing. Mr. T. W. Lister has similarly helped over the nursing of ophthalmic cases, and Dr. Theodore Thompson on blood culture and blood pressure. Dr. P. Fildes has contributed some information on syphilis and salvarsan, which, besides containing a sentence made very misleading by two errors in punctuation, is also open to objection on the score of less pardonable inaccuracies. Miss Lückes' own remarks on the subject of this disease are also not quite so admirable as is most of her teaching. A captious critic might also select for comment the paragraph in which it is insisted that sterilised materials alone must be used for padding splints. But in general it may be safely said that implicit faith may be placed in everything laid down in this book for the training and instruction of a nurse. If there were fewer quotations of poetry and fewer italicised words in the text, we cannot help thinking that from a purely literary standpoint the book would be greatly improved.

Standard Prescriptions for Insurance Practice. Compiled by C. H. Gunson, M.B., Ch.B. (London: The Scientific Press, Ltd. Price 1s.)

The prescriptions in this neat vest-pocket volume are wholly admirable from the point of view of practical usefulness for Insurance work. Such a compilation will save trouble to the doctors and the chemists in many ways, while the patients themselves, it may even be surmised, will fare perhaps more sumptuously than before. The list of mixtures which holds the place of honour in the book is a representative one, containing the usual run of ingredients still in vogue, both active and inert. In passing we may note that the only safe path for the compilers of these aids to prescribing is for them to stick to the abbreviations used in rapid writing. Besides the mixtures there is a useful collection of lotions, liniments, tablets, powders, drops, and other special formulæ; this makes the booklet of greater practical value. The prescriptions for children have no age assigned—an omission which is regrettable. As a formulary it is decidedly the best we have seen since the Insurance Act stimulated their production.

Essentials of Physiology. By F. A. Bainbridge, M.D., F.R.C.P., D.Sc., and J. Ackworth Menzies, M.D. With 134 illustrations. (London: Longmans, Green and Co., 1914. Price 10s. 6d. net.)

This new text-book on physiology for medical students is uniform with Schäfer's "Histology" and Halliburton's "Chemical Physiology," both well-known types of the "Essentials" series. The object of the authors has been to cater for the requirements of the student preparing for a pass examination only; for this purpose the book is completely suitable. The fundamental facts and principles of physiology are set out in a straightforward and easily assimilable style, without undue compression or any pretence of cramming methods. In fact, the pages require careful reading if all the excellent matter therein is to be properly appreciated, as may be gathered from the fact that the book runs to 417 pages of rather close print. As a text-book this new venture should achieve success by virtue of its convenient compactness and its happy exposition of physiological facts rather than by any claims to originality. Like many of the illustrations, not a few pages appeal to the reader as familiar friends; yet, on the other hand, there are many improved figures for which the modern student should be grateful, as well as for the simpler and more "learnable" explanations which are now prepared for his edification. In view of the transitional state of anatomical nomenclature it is well that the authors have not yet discarded the old names; they have, however, inserted the Basle nomenclature in brackets where desirable. Doubtless this text-book will have a vogue in the University of Durham, to which both the authors are attached, but it is safe to prophesy that the book will find acceptance in many other colleges of medicine.

